# The Role of Virtual Reality in Childhood Obesity Treatment: A Narrative Review

**DOI:** 10.3390/ijerph22020195

**Published:** 2025-01-29

**Authors:** Qutaibah Oudat, Sarah E. Messiah, Alia Dawlat Ghoneum

**Affiliations:** 1 Department of Population Health, College of Nursing, University of Cincinnati, Cincinnati, OH 45221, USA; 2 Department of Kinesiology and Public Health, Bailey College of Science and Mathematics, California Polytechnic State University, San Luis Obispo, CA 93407, USA; 3 Peter O’Donnell Jr. School of Public Health, University of Texas Southwestern Medical Center, Dallas, TX 75390, USA; sarah.messiah@utsouthwestern.edu; 4 Department of Family Medicine, East Carolina University, 101 Heart Drive, Greenville, NC 27834, USA; ghoneuma23@ecu.edu

**Keywords:** virtual reality, review, childhood obesity, obesity treatment, lifestyle treatment

## Abstract

Childhood obesity is a critical public health issue linked to long-term complications like type 2 diabetes and cardiovascular disease. This narrative review aimed to examine the effectiveness of virtual reality (VR)-based interventions in supporting key components of obesity treatment—physical activity (PA), nutrition education, and behavior change—particularly in children under 12. Using the intensive health behavior and lifestyle treatment (IHBLT) approach, we synthesized findings from studies published between 2014 and 2024. Of the studies reviewed, only one study met inclusion criteria, showing that VR-based interventions effectively increased light-intensity PA and reduced sedentary behavior in children, though these effects were not sustained long-term. The scarcity of studies limits the generalizability of these findings, emphasizing the need for caution in interpretation. Our review highlights a significant gap in the literature regarding VR’s role in childhood obesity treatment. Future research should explore the efficacy of VR in promoting PA, enhancing nutrition education, and supporting sustained behavior changes. Expanding this evidence base is vital for developing accessible and engaging interventions tailored to young children. Addressing this gap could enhance obesity treatment options, offering innovative and effective strategies to improve health outcomes in this vulnerable population.

## 1. Introduction

Childhood obesity remains a pressing public health concern in the United States, affecting approximately one in five children [1,2]. Obesity in children increases the risk of several health conditions, including metabolic syndrome, type 2 diabetes, heart disease, 13 types of cancer, and liver and kidney diseases, that can persist into adulthood and later life [3]. The American Academy of Pediatrics guidelines recommend several options to manage and treat obesity, depending on the severity of obesity and eligibility [4,5]. These options include intensive health behavior and lifestyle treatment (IHBLT) for children under 12 years, with consideration of weight loss medications for those aged 12 and older, and bariatric surgery as part of the treatment plan for those aged 13 and older [6]. IHBLT is multi-component and consists of nutrition education, physical activity, and behavioral change support (behavior change technique (BCT)). It involves a minimum of 26 h of face-to-face (FTF) treatment, over a period of 3 to 12 months. Typically, it is delivered by trained healthcare professionals with the active involvement of a parent or caregiver [4,5].

However, despite its effectiveness, IHBLT has several challenges, including a significant time commitment from parents/caregivers, lack of community resources, being resource-intensive, and limited availability and accessibility [4,7]. For instance, participants, particularly in rural or underserved areas, may unintentionally be excluded from obesity prevention programs due to the lack of transportation or high travel costs to IHBLT sessions. These challenges underscore the need to explore alternative modalities to support delivering the IHBLT and obesity treatment programs.

Novel modalities, such as virtual reality (VR), have garnered significant attention in the past few decades in both clinical practice and research, especially during and after the COVID-19 pandemic [8,9,10]. Recent studies have shown that VR holds a promising potential for promoting sustainable behavior changes, offering an inexpensive and largely accessible tool to engage participants from diverse and remote populations [11,12]. The nature of VR can mimic real-world scenarios, allowing for the dynamic, collaborative, and interactive engagement of participants. VR can stimulate cognitive, emotional, and physiological responses to create engaging experiences [13]. For example, VR offers an immersive experience where participants can move, see, hear, and interact with their virtual environment, and in some cases, even sense and touch objects through the use of haptic feedback technology [14,15].

Studies on VR’s potential for obesity treatment or weight management, particularly in adults, have shown that VR-based interventions can significantly support weight loss, enhance body image perceptions, and improve self-efficacy in patients with obesity [16,17,18,19]. Findings of these reviews have also demonstrated that VR can simulate realistic environments, such as grocery shopping or social situations, to help participants safely confront real-life scenarios at their own pace, practice healthier habits, and develop better emotional and behavioral responses toward food. For instance, the VR environment allows participants to respond to triggers that would typically lead to unhealthy behaviors, such as overeating or making poor food choices, without the fear of real-life consequences [16,17,18,19]. This can, in turn, enable participants to develop and strengthen their ability to handle similar situations in real-life scenarios and enhance their ability to make sustained behavioral changes. Despite its promising outcomes among adults, current reviews have not explored the role of VR use in obesity treatment in children. Guided by the IHBLT regimen, this narrative review aimed to review and synthesize current evidence regarding the effectiveness of VR-based intervention in enhancing PA and nutrition education and supporting behavior change in children aged under 12. The primary question for this review was as follows: in children under 12, how do VR-based interventions compare to non-VR or standard interventions in their impact on (1) PA, (2) nutrition education, and (3) behavior change support? Understanding the implications of VR in obesity treatment in children can significantly contribute to developing more effective and engaging interventions. VR offers unique opportunities to simulate real-world environments, making it easier for children to adopt healthier behaviors in a controlled, interactive setting. This review also contributes to the growing body of knowledge about VR use in medical research and practice, providing insights into how VR can enhance motivation, increase physical activity, and offer tailored support for behavior change in pediatric obesity management.

## 2. Materials and Methods

This is a narrative review aimed at systematically synthesizing the current literature regarding the effectiveness of VR-based intervention compared to non-VR or standard interventions for obesity treatment in children aged under 12. The expanded PRISMA guidelines [20] were used to guide the conduct and reporting of this review to ensure methodological rigor and transparency, offering a structured framework that enhances the clarity and reproducibility of this review.

### 2.1. Eligibility Criteria

To be included, studies had to meet the following inclusion criteria: (1) full-text experimental design articles (randomized controlled trials (RCTs) or quasi-experimental), (2) written in English, (3) involving children aged under 12, and (4) published between January 2014 and September 2024. This timeframe can help to capture the latest studies that reflect the current state of VR technology and its applications in obesity treatment, capturing the most up-to-date and relevant findings. The search terms were derived from the IHBLT regimen for obesity treatment. Additionally, studies were included if they reported results addressing the effectiveness of VR-based intervention to improve physical activity, nutrition education, or behavior change support in children under 12 years old. Further, studies had to involve at least one primary caregiver/parent and a child less than 12 years old.

Studies were excluded if they involved children or caregivers/parents with health conditions or studies that included children above 12 years old. Exclusion criteria also included the following: nonexperimental studies, books, presentations, conference papers or statements, dissertations and theses, expert opinions, and newspaper articles.

### 2.2. Information Sources

Three electronic databases, PubMed, CINAHL Plus with Full Text, and APA PsycINFO (via EBSCOhost), were used to identify eligible articles. Websites and search engines were also browsed for supplementary articles, including Google Scholar (scholar.google.com (accessed on 20 January 2025)) and ResearchGate (https://www.researchgate.net (accessed on 20 January 2025)). This search was conducted by the main author of this review (QO), who has expertise in obesity prevention and treatment. No organizations or manufacturers were directly contacted; however, individual researchers with expertise in the area were consulted to identify additional relevant studies. Reference lists of studies included in this review and systematic reviews on related topics were examined for additional references.

### 2.3. Search Strategy

Comprehensive search terms were used to capture relevant studies. The following search terms were used in each database: “parents” OR “caregivers” OR “mother” OR “father” OR “parent” OR “family” OR “families” AND “children” OR “adolescents” OR “youth” OR “child” OR “teenager” OR “pediatric” OR “paediatric” OR “kids” AND “physical activity” OR “exercise” OR “fitness” OR “physical exercise” OR “sport” OR “walking” OR “cycling” AND “behavioral change” OR “behavior modification” OR “lifestyle change” OR “behavior change techniques” OR “setting” OR “self-monitoring” OR “stimulus control” OR “cognitive restructuring” OR “stress management” OR “problem-solving” OR “social support and information” AND “nutrition education” OR “nutrition knowledge” OR “healthy eating education” OR “nutrition education intervention” OR “diet counseling”.

### 2.4. Selection Process

Articles retrieved were imported to the EndNote 20 desktop version [21] and duplicated records were removed before the screening process started. Two reviewers (QO and AG) independently performed the screening processes to ensure rigor and avoid bias in selecting studies. The screening process involved two phases: (1) title and abstract and (2) full text. Ongoing meetings were held to discuss the selected articles and resolve disagreements. No articles required translation as all retrieved records were in English.

### 2.5. Data Items (Outcomes)

Guided by the HBLT regimen for obesity treatment, we sought data in this review for the following areas: (1) physical activity, (2) nutrition education, and (3) behavior change support. Each study included in this review was screened and several datapoints were collected and organized into a comprehensive table, as suggested by Melnyk and Morrison-Beedy (2012) [22]. This table included information, such as study reference and country, participants’ characteristics and research setting, and intervention characteristics, including the duration, study design and purpose, technology used, and main results. Details are provided in Supplemental Table S1 [23].

## 3. Results

**Figure 1:**
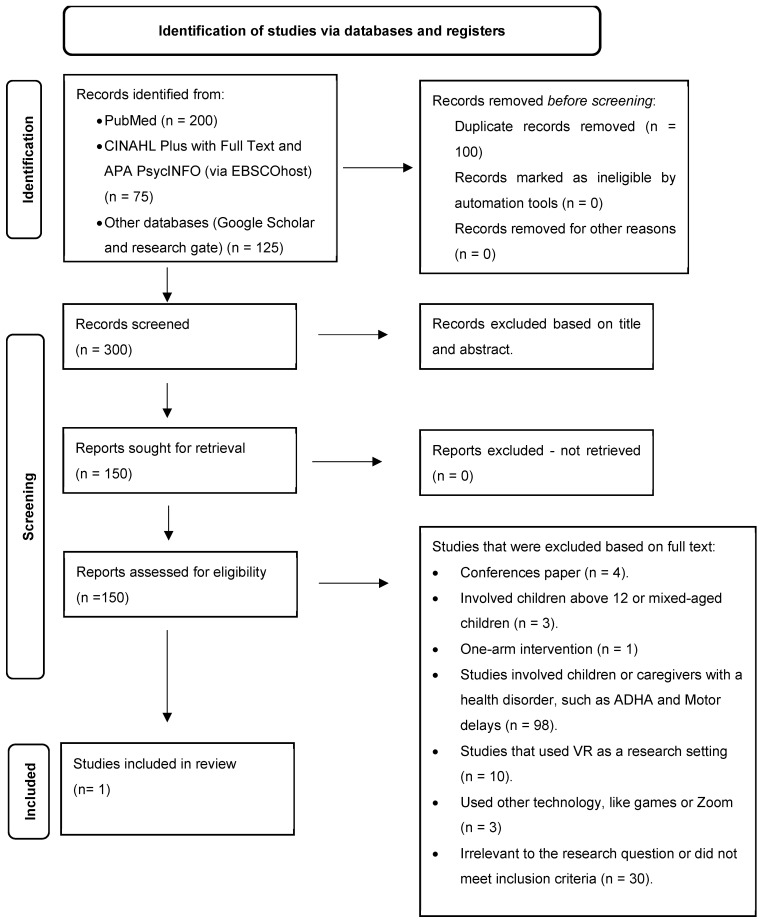
PRISMA flow diagram.

The PRISMA flow diagram (Figure 1) was used to present the search and selection process [20]. Initially, we retrieved 400 records from the database searches and other sources. After removing 100 duplicates, 300 records remained for title, abstract, and full-text screening. Of these, 250 records were excluded during the initial screening phase based on title and abstract, and an additional 49 records were excluded following full-text review. Retrieved records were excluded for several reasons, including irrelevance to the review’s objectives (e.g., studies not focused on VR interventions, conference papers, one-arm intervention, did not meet inclusion criteria, or included children or caregivers/parents with a health disorder). In total, one study was eligible to be included in this review. Details are provided in Supplemental Table S1.

### 3.1. Impact of VR on Physical Activity

Regarding physical activity, the majority of the studies retrieved (n = 98) addressed the effectiveness of VR-based interventions to treat or manage physical activity in children with specific health conditions, such as movement disorders or ADHD. For example, a study by Shema-Shiratzky et al. (2019) [24] assessed the feasibility and efficacy of combined motor–cognitive training using VR to enhance behavior, cognitive function, and dual-tasking in children with ADHD. The study found significant improvements in social behavior, executive function, memory, and gait regularity, with long-term benefits maintained in memory and executive function [24]. Additionally, a pilot study by Polechoński et al. (2020) [25] in Poland included a single experimental group. The study aimed to assess both the attractiveness and the intensity of physical exercise when using active video games (AVGs) in immersive virtual reality (IVR) settings. The study sample (n = seven boys and four girls) engaged in controlled laboratory sessions at a clinic in Katowice, where they engaged with two different AVGs: *Core Defense* and *Travar Training OPS*. Each game session lasted 15 min, with a 30 min rest period between the two games. The findings from this study indicated that both AVGs induced high-intensity physical activity, meeting the World Health Organization’s (WHO) recommendations for health benefits [26]. Specifically, *Travar Training OPS* resulted in a higher exercise intensity (83.3% HRmax) compared to *Core Defense* (77.4% HRmax). The children’s perceived exertion corresponded with the physiological data, suggesting that the VR-based AVGs provided a substantial physical challenge. Additionally, all participants found the games appealing, and a significant majority expressed a preference for AVGs over conventional video games and traditional physical activities. Notably, 91% of the children preferred AVGs to conventional video games, 73% were willing to replace conventional games with AVGs, and 64% favored AVGs over traditional physical activities [25].

Of the studies retrieved, one study met the eligibility criteria. Despite the limited number of studies, the findings offer valuable insights into the potential benefits and challenges of employing VR technology to promote physical activity in young children aged under 12. In the US, Ahn et al. (2024) conducted a cluster randomized controlled trial that examined the efficacy of the “Virtual Fitness Buddy” (VFB) ecosystem. This precision health intervention was designed to increase physical activity and decrease sedentary behavior among children aged 6 to 11 years. The study involved a larger sample of 303 children from 19 elementary schools and YMCA branches in Metro Atlanta, Georgia [27]. Approximately 40% of the children had either overweight or obesity (BMI percentile >85th percentile adjusted for age and sex). The intervention was grounded in Self-Determination Theory, emphasizing autonomy, competence, and relatedness to foster intrinsic motivation for behavior change. In the intervention group, children interacted with a custom virtual dog through a mixed-reality kiosk equipped with motion-tracking technology. This virtual pet provided real-time feedback on the children’s physical activity progress. Parents received updates via a mobile app, enabling them to offer encouragement and support. The control group, in contrast, used a computer system to set physical activity goals without any interaction with a virtual pet or parental support. The intervention lasted six months for each cohort, and physical activity levels and sedentary behavior were measured using Fitbit activity monitors for daily tracking and Actigraph accelerometers worn for one-week periods at baseline, three months, and six months. Results showed that the VFB ecosystem was effective in reducing sedentary behavior and increasing light-intensity physical activity in the short term, particularly during the three-month assessment. Children who had lower baseline levels of moderate-to-vigorous physical activity and those who owned real dogs showed greater benefits from the intervention. The involvement of parents, who could actively support and encourage their children’s physical activity efforts, appeared to enhance the intervention’s effectiveness. However, the positive effects observed at three months were not sustained at the six-month follow-up [27].

Overall, despite the limited evidence, results demonstrated that immersive and interactive technologies can increase engagement, enjoyment, and motivation to participate in physical activity, which is particularly important for children who are obese or have low baseline activity levels. The results also emphasize the importance of parental involvement and personalized feedback in supporting behavior change. However, the limited number of studies and their methodological constraints, such as small sample sizes, short duration, lack of control groups, and challenges in sustaining engagement, underscore the need for further research.

### 3.2. VR-Driven Nutrition Education

Our comprehensive search yielded no studies that addressed the effectiveness of VR-based interventions to promote the nutrition awareness of children. However, a limited number of retrieved and screened studies used various virtual ways to promote nutrition education, such as games or educational sessions delivered via Zoom [28,29,30]. Additionally, four conference reports discussed the potential effectiveness of VR interventions in supporting healthier food choices and enhancing nutrition awareness learning systems for children [31,32,33,34]. Despite not meeting the eligibility criteria, these studies and reports suggest promising effects of VR-based intervention in promoting healthy food consumption and increasing engagement and motivation to learn about healthy nutrition. VR can be an immersive tool that enhances experiential learning, allowing children and their parents to participate actively in realistic simulations of healthy eating scenarios. This interactive environment can foster effective learning experiences, where families can understand the consequences of certain food choices and practices on their young children. Overall, there remains a significant gap in the existing literature regarding the effects of VR-based nutrition education on children’s dietary behaviors and food choices.

### 3.3. Facilitating Behavior Change Through VR

Behavior change refers to a comprehensive process of altering someone’s actions, habits, or routines to achieve a specific goal, usually in the context of improving health and well-being. Specifically, the term “behavior change” in this review referred to the use of behavior change techniques (BCTs), such as goal setting, self-monitoring, and reinforcement—which are often applied to support sustained engagement in both physical activity and healthier dietary behaviors [35,36].

In our review, we searched for experimental studies that addressed the role of VR in supporting the behavioral change of children with obesity, especially ones that used BCTs. However, our comprehensive search indicated that no studies have yet addressed the implication of VR-based intervention to facilitate behavioral change in children with obesity.

## 4. Discussion

Guided by IHBLT, the purpose of this narrative review was to synthesize the existing literature regarding the effectiveness of VR-based intervention compared to non-VR or standard interventions for obesity treatment in children aged under 12. The results indicated that VR technology can hold promising outcomes, but limited studies addressed its impact on treating obesity in children under 12.

Regarding PA, our review has yielded one study showing that the VFB intervention effectively reduced sedentary behavior and increased light-intensity physical activity in young children within the first three months, though the effects were not sustained at the six-month follow-up [27]. Existing reviews have primarily focused on adult populations (≥18 years), which consistently demonstrated that VR positively influences physical activity by enhancing enjoyment and motivation [37,38,39]. However, while these findings are promising, they are still limited, particularly when considering their applicability to children. Thus, the applicability of these results to children should be taken with caution. Therefore, further research is essential to evaluate the effectiveness of VR-based interventions in promoting and creating sustained PA in children.

This review has also examined the effectiveness of VR-based interventions to promote nutrition education in children under 12 years old. While no studies were found, the studies retrieved, including conference papers, indicated that virtual teaching sessions, for example, via Zoom, could positively impact nutrition awareness, especially for parents/caregivers. Additionally, the conference papers retrieved call for more studies to address the potential impact of VR on nutrition education in children. Existing studies have mainly examined various interventions delivered using VR immersive educational environments to particularly help adult populations (≥18) understand healthy eating. Findings demonstrated that VR can be a novel tool for the education and rehabilitation of nutrition [31,40,41]. These studies have consistently highlighted the educational benefits of VR in nutrition, with promising results in improving dietary behavior and enhancing user engagement through realistic, interactive experiences. However, studies have not yet addressed the effects of VR use on enhancing food awareness among children. Future studies should focus on exploring how VR-based interventions can be tailored to suit the developmental and cognitive abilities of children under 12. Specifically, these studies should aim to investigate whether VR can effectively teach children about nutrition concepts such as food groups, portion sizes, and the importance of balanced diets in an engaging and age-appropriate manner. Moreover, there is a need to assess whether these interventions can influence children’s food choices and eating behaviors long-term. Future research should also explore the role of parental involvement in such interventions, as parents and caregivers are key in shaping children’s dietary habits. Understanding how VR can complement traditional educational tools, such as in-class lessons or family-based nutrition education, will be crucial in determining its broader applicability and effectiveness.

Finally, existing studies have consistently found that BCTs have been positively associated with food consumption and PA, especially in adults [42,43]. The literature is still limited regarding the application of VR as a tool to administer lifestyle interventions to treat obesity. Our review did not find studies that examined the effectiveness of VR-based interventions to support behavioral change to treat obesity in children aged under 12. Future studies should focus on the development and evaluation of VR programs that incorporate behavior change techniques, such as goal setting, self-monitoring, and feedback. Moreover, investigating the long-term effects of VR interventions on maintaining behavior changes and reducing the risk of obesity in children will be critical for understanding the full potential of this approach.

### 4.1. Policy Recommendations

The findings of this review revealed that the application of VR in obesity treatment among children remains limited, with insufficient evidence to support its widespread implementation as a primary intervention. While preliminary studies suggest potential benefits, further research is essential to establish the efficacy, sustainability, and scalability of VR-based interventions before they can be confidently integrated into public health and clinical practice. Based on the current state of evidence, we propose recommendations to guide future research and policy development. Future efforts may prioritize rigorous RCTs and pilot programs to evaluate VR’s impact on physical activity, nutrition education, and behavior change. Tailored, age-appropriate interventions that address the developmental needs of children under 12 and incorporate ethical safeguards, such as screen time limits and data privacy protections, are critical. Parental involvement and accessibility for underserved communities may also enhance effectiveness and equity. We recommend that VR should currently be considered a supplementary tool rather than a primary modality. Policymakers may adopt a cautious approach, emphasizing further research and cross-sector collaboration to ensure the safe, effective, and equitable application of VR in childhood obesity prevention. Table 1 summarizes the gaps in the literature, highlights possible implications, and proposes recommendations to address these gaps in future research.

### 4.2. Strengths and Limitations

Unlike other reviews [17,18,19], this review is among the first to comprehensively explore the effectiveness of VR-based interventions for addressing obesity in children. It synthesized evidence across studies that examine VR’s ability to engage children in physical activity, provide nutrition education, and support behavior change, addressing critical gaps in current interventions aimed at this age group.

The limitations identified in this review are common and consistent with those encountered in many other systematic reviews in the field. A key limitation of this review was the small number of studies that met the inclusion criteria, which could reflect the limited evidence currently available in this field. While this scarcity highlights the need for further investigation, it also impacts the robustness and generalizability of the findings. As a result, the conclusions drawn from this review should be interpreted with caution. This limitation aligns with those reported in similar reviews, such as Al-Rasheed et al. (2022) [17], which also emphasized the scarcity of high-quality studies in this area. Furthermore, this review was limited to English-language full-text articles retrieved from three electronic databases: PubMed, CINAHL Plus with Full Text, and APA PsycINFO. This restriction may have led to the exclusion of relevant studies published in other languages or indexed in additional databases, potentially resulting in an incomplete representation of the global research landscape on VR interventions for treating pediatric obesity. The issue of potential language bias, as noted by Rumbo-Rodriguez et al. (2020) [19], further underscores the importance of interpreting the findings with caution in light of these limitations.

## 5. Conclusions

Guided by the IHBLT regimen, this review aimed to evaluate the effectiveness of VR-based interventions compared to non-VR or standard approaches for treating obesity in children under 12. While the existing literature demonstrates the significant potential of VR to provide cost-effective and more accessible interventions that can reach a more diverse population, our findings indicate that the application of VR in obesity treatment, particularly among children, is still limited and requires further investigation. Understanding the application of VR in this context is crucial, as it offers unique opportunities to engage children and their parents/caregivers in interactive and immersive experiences that can contribute to the efforts of treating obesity in children.

## Figures and Tables

**Table 1:** Summary of the gaps in the literature and recommendations for future research.

Identified Gap	Possible Implications	Recommendation
Limited studies on VR-based interventions for obesity treatment in children under 12	The scarcity of VR-based studies for pediatric obesity limits evidence-based implementation.	Conduct more RCTs with larger sample sizes and control groups to evaluate effectiveness. Incorporate longer follow-up periods to assess sustained outcomes.
Lack of research on VR-driven nutrition education for young children	Insufficient evidence on VR’s role in enhancing nutrition education hinders its application in this area.	Develop and test VR-based nutrition education programs tailored to the developmental and cognitive abilities of children under 12. These programs should aim to teach nutrition concepts engagingly and interactively. Research should assess the impact of these interventions on children’s dietary behaviors, food choices, and long-term eating habits. Including parental involvement could enhance the effectiveness of these interventions.
Absence of studies on VR facilitating behavior change using behavior change techniques (BCTs) in children with obesity	Current VR interventions lack integration of BCTs proven effective in behavior modification.	Design VR interventions that incorporate established behavior change techniques, such as goal setting, self-monitoring, feedback, and social support, specifically for children with obesity. Evaluate the effectiveness of these interventions in facilitating behavior change and weight management. Studies should consider unique motivational factors and engagement strategies suitable for children under 12.
Short-term effectiveness of VR interventions not sustained long-term	Declines in engagement or novelty effects reduce long-term intervention success.	Conduct longitudinal studies to assess sustained outcomes. Implement reinforcement strategies and combine VR with other supportive measures.
Limited understanding of parental involvement in VR interventions for children	Parental roles in VR interventions remain underexplored, limiting their potential effectiveness.	Investigate how parental engagement enhances outcomes through joint participation and reinforcement. Design VR interventions that educate and involve parents.
Need for tailored VR content suitable for young children	Generic VR content may not meet developmental needs of young children.	Create age-appropriate, engaging, and accessible VR content. Assess usability and acceptability among children and families for optimal implementation.
Potential barriers to access and equity in VR interventions	Cost and accessibility challenges limit widespread adoption of VR interventions.	Develop affordable VR solutions and implement community-based programs. Use the RE-AIM framework to ensure interventions are equitable and inclusive.

## Supplementary Materials

The following supporting information can be downloaded at https://www.mdpi.com/article/10.3390/ijerph22020195/s1: Supplemental Table S1: Characteristics of the included studies.
