# Supplementary material for: The Role of Virtual Reality in Childhood Obesity Treatment: A Narrative Review
Source: Int J Environ Res Public Health. 2025 Jan 29;22(2):195. doi: 10.3390/ijerph22020195 (PMC11855200; doi:10.3390/ijerph22020195)
Supplement: Supplementary file 1 [file ijerph-22-00195-s001.zip › ijerph-3384133-supplementary.pdf]

Supplemental Table S1. Characteristics of the Included studies.

Author, Year [References], Country, Study Design, Theoretical Framework	Purpose, Sample Characteristics, Setting	Number of groups, Components and Duration (Intervention and Control Group)	Measures and Follow-Up Period*	Technology	Key Results	Main Limitations
**Theme** 1. Physical Activity						
(Ahn et al., 2024)<br><br>US<br><br>Cluster randomized controlled trial (RCT)<br><br>Self-Determination Theory (focus on autonomy, competence, and relatedness to foster intrinsic motivation for behavior change).	• **Purpose**: To test the efficacy of the Virtual Fitness Buddy (VFB) ecosystem, a precision health intervention, in increasing physical activity (PA) and decreasing sedentary behavior among children aged 6–11 years.<br>• 303 children aged 6–11 years old. Majority of primary caregivers were female, and children were majority boys. 40% of children had a BMI > 85.<br>• **Setting**. 19 elementary schools and YMCA branches in Metro Atlanta, Georgia.	• Two groups: (1) Treatment group (interacted with the Virtual Fitness Buddy ecosystem), (2) Control group (used a computer system to set PA goals without feedback from the virtual dog or their parents).<br>• **Treatment Group**: Children interacted with a custom virtual dog via a mixed reality kiosk that provided feedback on PA progress. Parents received real-time updates and could offer support.<br>• **Control Group**: Children set PA goals on a computer without the virtual dog or parental support.<br>• **Duration**: 6 months for each cohort.	• PA and SB were measured using both Fitbits (daily tracking) and Actigraphs (worn for 1-week periods at baseline, 3 months, and 6 months). The primary outcomes were PA intensity levels (light, moderate, vigorous) and sedentary behavior changes.	• Wearable Fitbit activity monitors, mixed-reality kiosk with motion-tracking for virtual dog interaction , and Actigraph accelerometers for additional PA validation.	• The Virtual Fitness Buddy ecosystem was effective in reducing sedentary behavior and increasing light-intensity PA in the short term, particularly after 3 months.<br>• Children with lower baseline moderate-to-vigorous PA benefited more, and children who owned real dogs also showed stronger effects.<br>• PA gains and sedentary behavior reductions were not sustained at the 6-month follow-up.	• Seasonality (winter months) may have contributed to the drop-off in PA during the second half of the intervention.<br>• Accessibility to the mixed-reality kiosks varied between schools, which may have affected engagement.<br>• Digital content was not updated throughout the study.
**Theme 2.** Nutrition Education						
No Studies Found						
**Theme 3.** Behavior Change Support						
No Studies Found						
